# Supplementary figures and images for: Vascular Niche Facilitates Acquired Drug Resistance to c-Met Inhibitor in Originally Sensitive Osteosarcoma Cells
Source: Cancers (Basel). 2022 Dec 15;14(24):6201. doi: 10.3390/cancers14246201 (PMC9776923; doi:10.3390/cancers14246201)

Figure 1 (c)

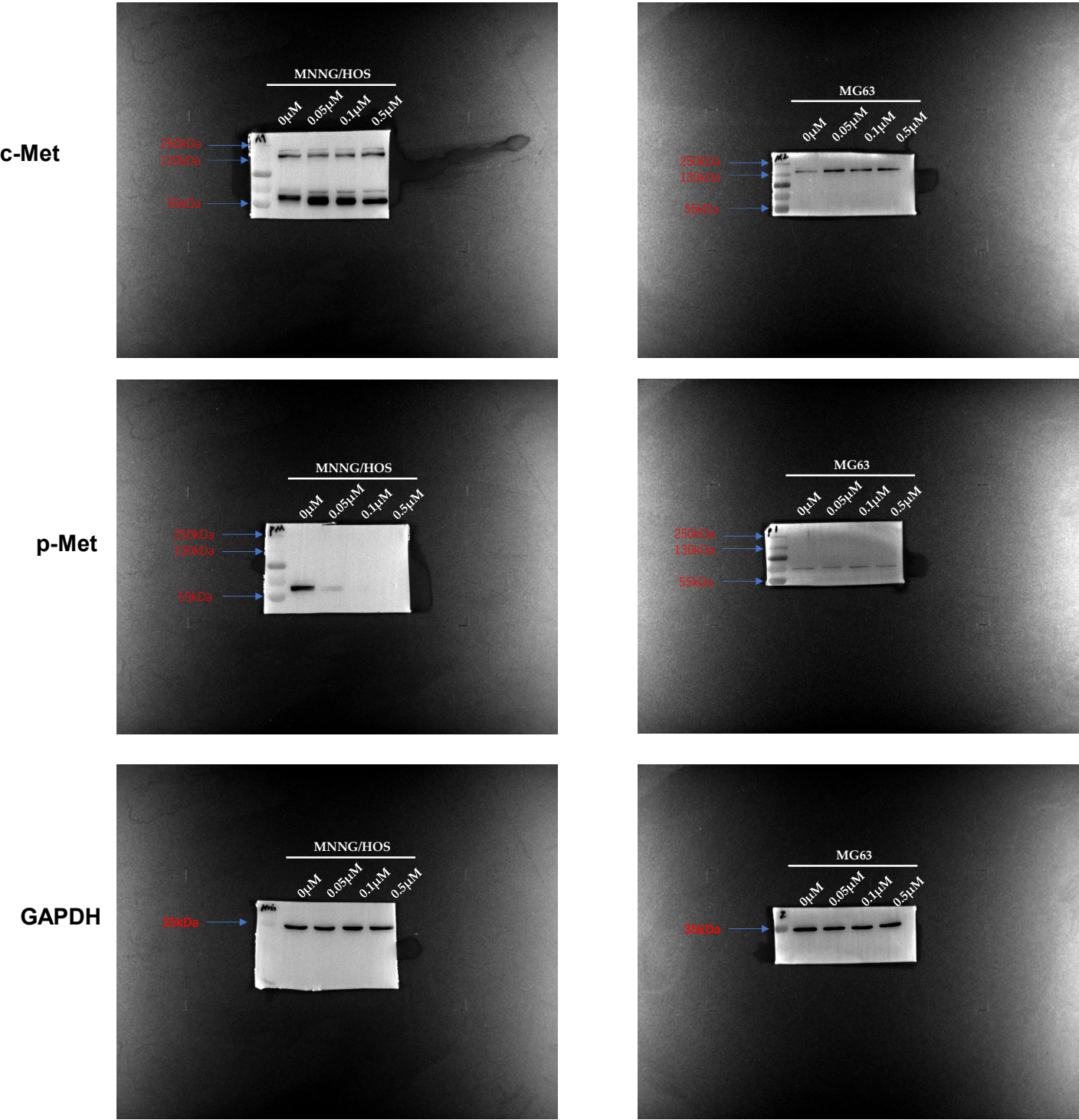

Figure 2 (b)

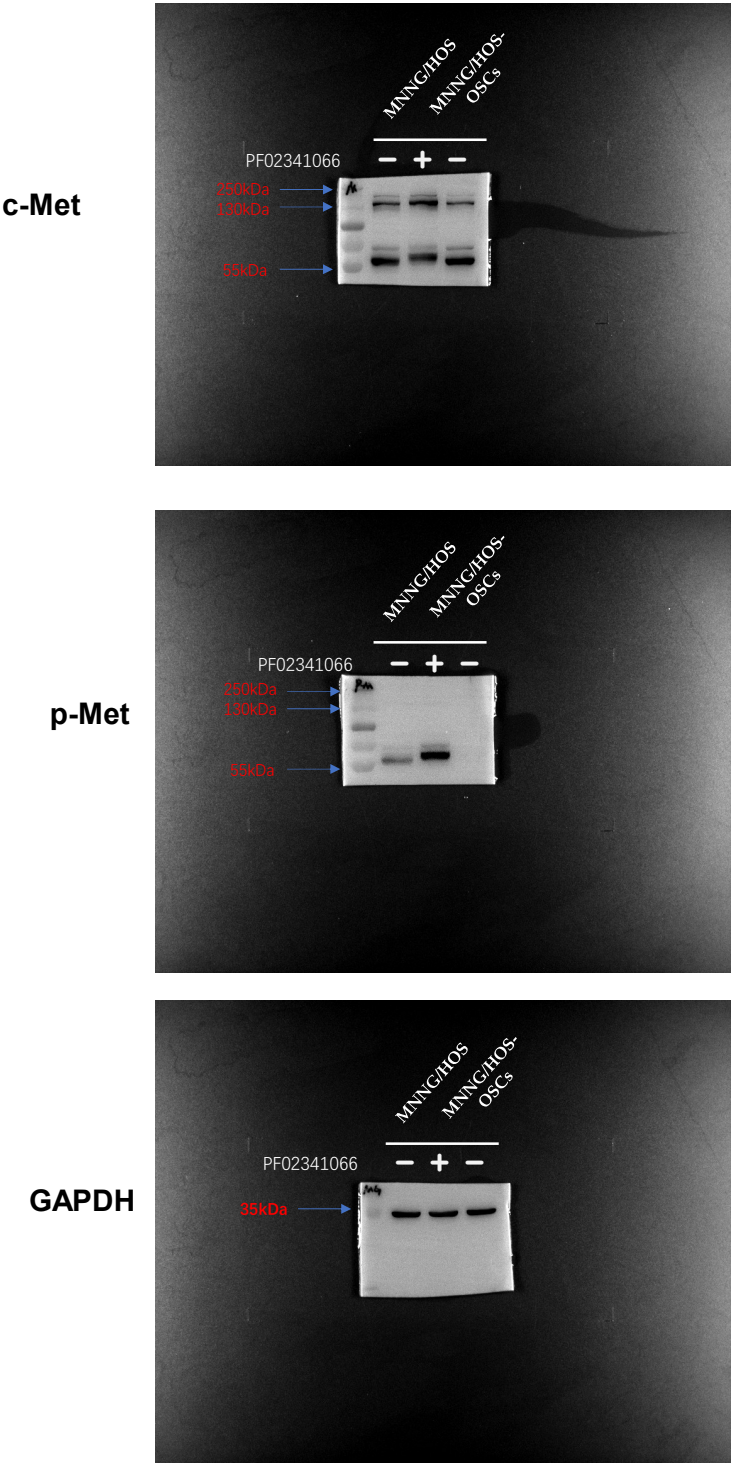

Figure 5 (c)

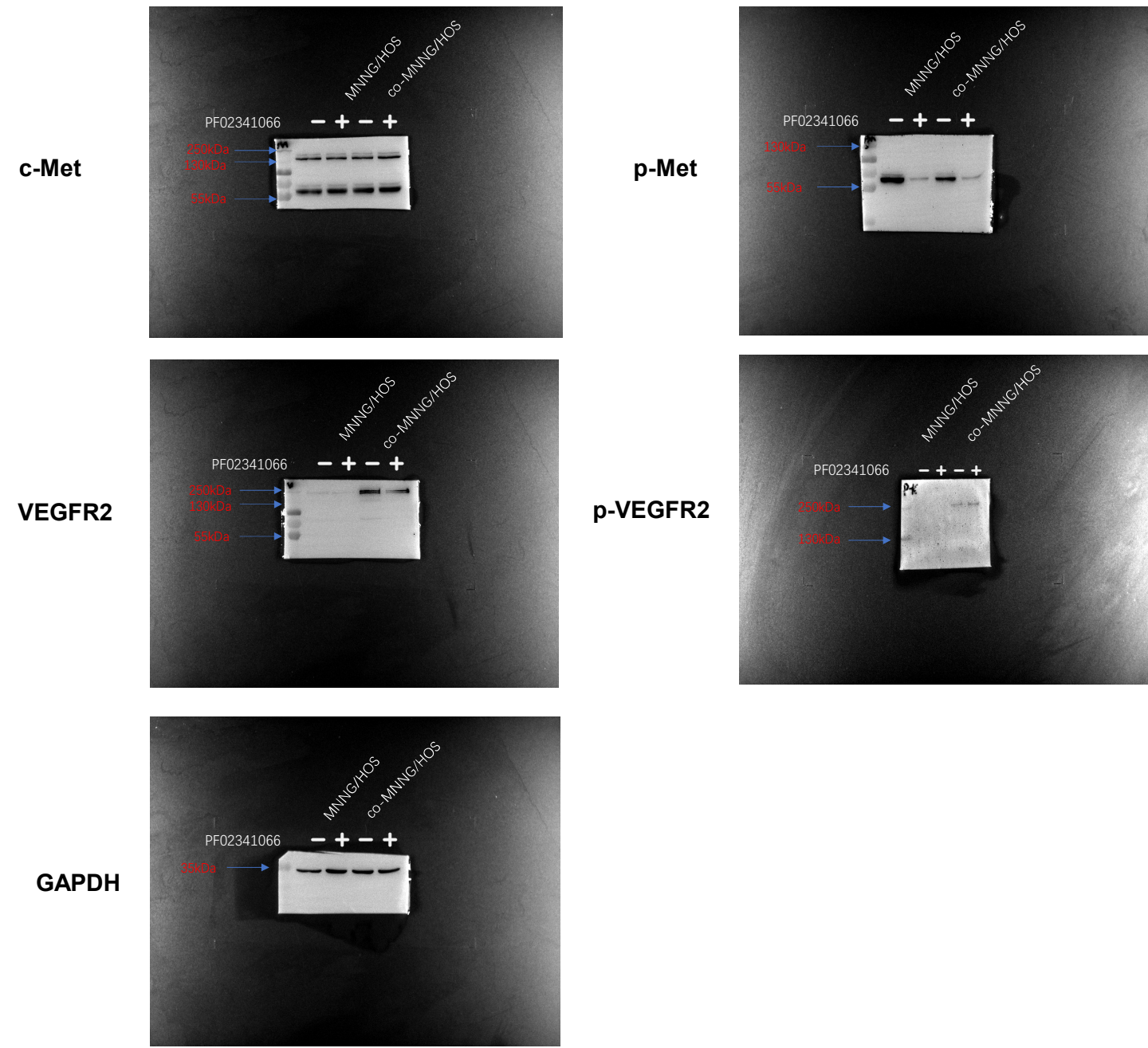

Figure 5 (g)

c-Met

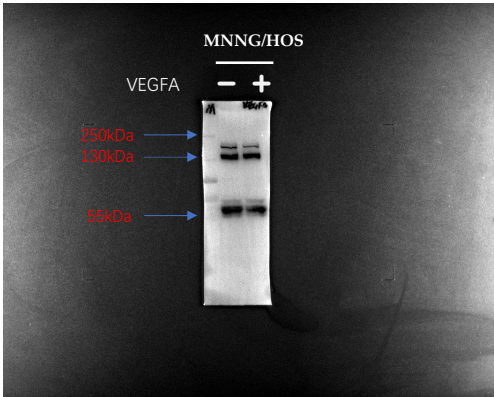

p-Met

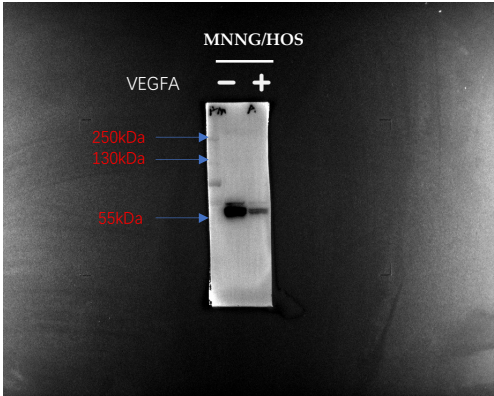

GAPDH

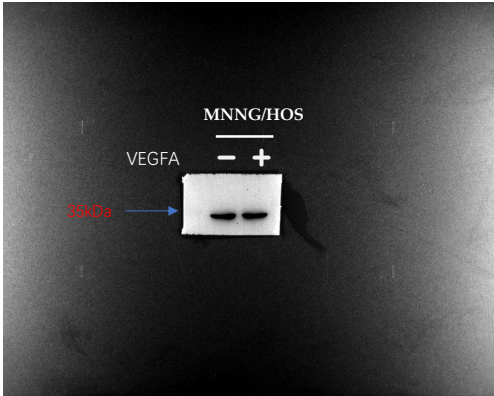

Figure 5 (h)

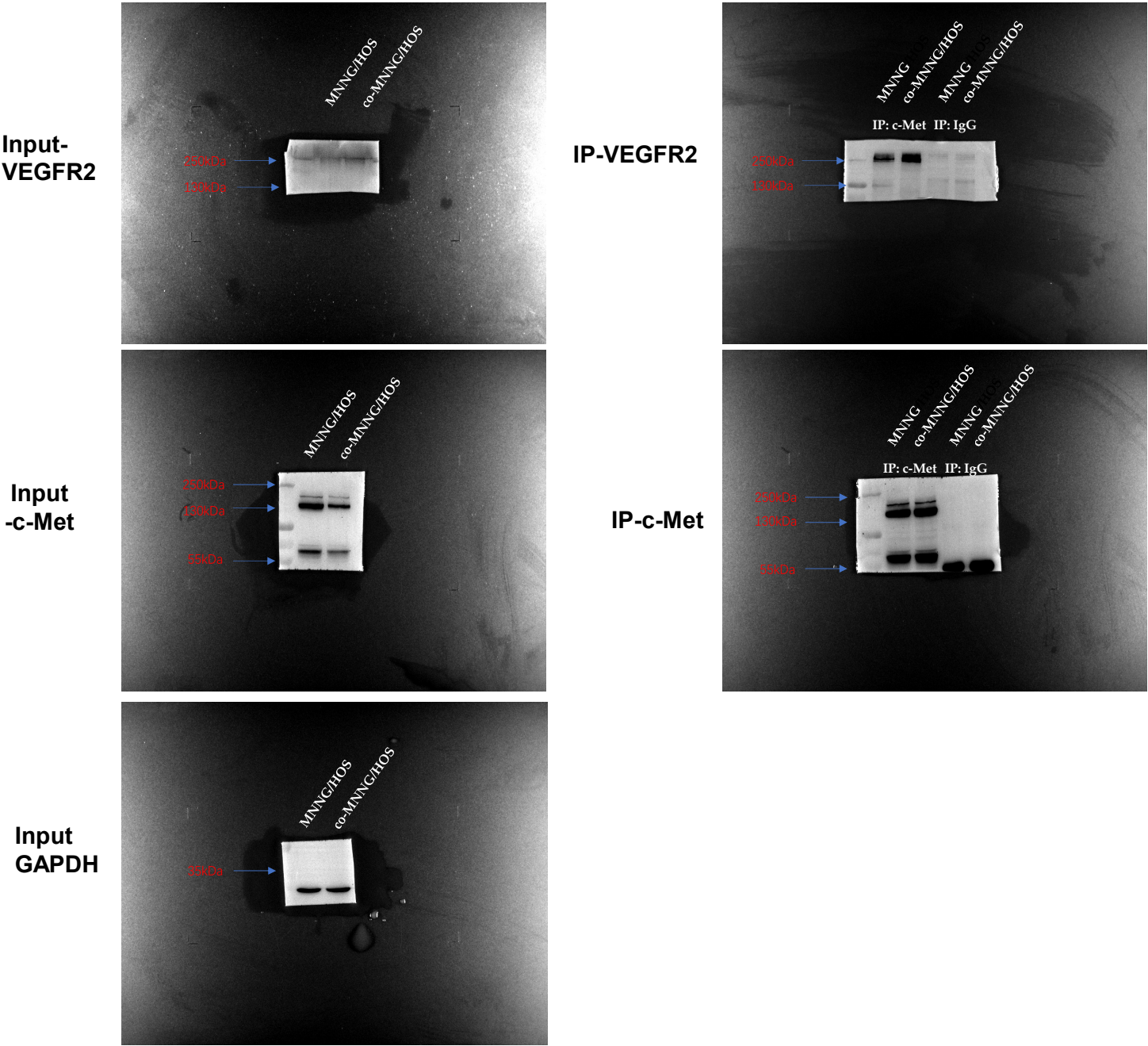

Figure 6 (b)

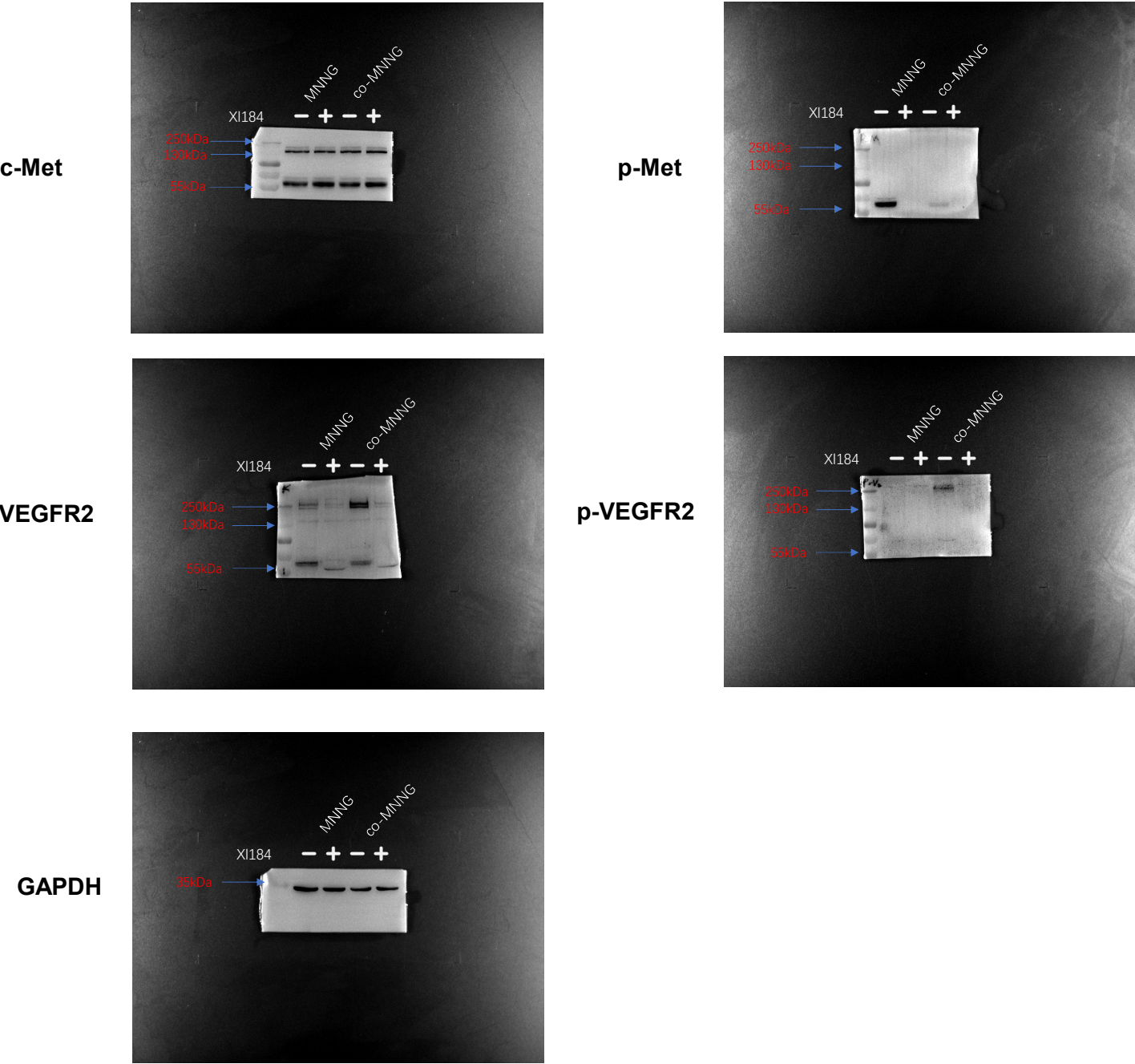

Supplement: Supplementary file 1 [file cancers-14-06201-s001.zip › cancers-2017614-supplementary.pdf]
